# *OptRAM*: *In-silico* strain design via integrative regulatory-metabolic network modeling

**DOI:** 10.1371/journal.pcbi.1006835

**Published:** 2019-03-08

**Authors:** Fangzhou Shen, Renliang Sun, Jie Yao, Jian Li, Qian Liu, Nathan D. Price, Chenguang Liu, Zhuo Wang

**Affiliations:** 1 Bio-X Institutes, Key laboratory for the Genetics of Developmental and Neuropsychiatric Disorders (Ministry of Education), Shanghai Jiao Tong University, Shanghai, China; 2 School of Life Sciences and Biotechnology, Shanghai Jiao Tong University, Shanghai, China; 3 Department of Biomedical Engineering, University of Michigan, Ann Arbor, Michigan, United States of America; 4 Institute for Systems Biology, Seattle, Washington, United States of America; CPERI, GREECE

## Abstract

The ultimate goal of metabolic engineering is to produce desired compounds on an industrial scale in a cost effective manner. To address challenges in metabolic engineering, computational strain optimization algorithms based on genome-scale metabolic models have increasingly been used to aid in overproducing products of interest. However, most of these strain optimization algorithms utilize a metabolic network alone, with few approaches providing strategies that also include transcriptional regulation. Moreover previous integrated approaches generally require a pre-existing regulatory network. In this study, we developed a novel strain design algorithm, named OptRAM (**Opt**imization of **R**egulatory **A**nd **M**etabolic Networks), which can identify combinatorial optimization strategies including overexpression, knockdown or knockout of both metabolic genes and transcription factors. OptRAM is based on our previous IDREAM integrated network framework, which makes it able to deduce a regulatory network from data. OptRAM uses simulated annealing with a novel objective function, which can ensure a favorable coupling between desired chemical and cell growth. The other advance we propose is a systematic evaluation metric of multiple solutions, by considering the essential genes, flux variation, and engineering manipulation cost. We applied OptRAM to generate strain designs for succinate, 2,3-butanediol, and ethanol overproduction in yeast, which predicted high minimum predicted target production rate compared with other methods and previous literature values. Moreover, most of the genes and TFs proposed to be altered by OptRAM in these scenarios have been validated by modification of the exact genes or the target genes regulated by the TFs, for overproduction of these desired compounds by *in vivo* experiments cataloged in the LASER database. Particularly, we successfully validated the predicted strain optimization strategy for ethanol production by fermentation experiment. In conclusion, OptRAM can provide a useful approach that leverages an integrated transcriptional regulatory network and metabolic network to guide metabolic engineering applications.

## Author summary

Computational strain design algorithms based on genome-scale metabolic models have increasingly been used to guide rational strain design for metabolic engineering. However, most strain optimization algorithms only utilize a metabolic network alone and cannot provide strategies that also involve transcriptional regulation. In this paper, we developed a novel strain design algorithm, named OptRAM (**Opt**imization of **R**egulatory **A**nd **M**etabolic Network), which can identify combinatorial optimization strategies including overexpression, knockdown or knockout of both transcription factors and metabolic genes, based on our previous IDREAM integrated network framework. OptRAM uses simulated annealing with a novel objective function, which can ensure a favorable coupling between the production of a desired chemical and cell growth. This strategy can be deployed for strain design of bacteria, archaea or eukaryotes. The other advantage of OptRAM compared with previous heuristic approaches is that we systematically evaluated the implementation cost of different solutions and selected strain designs which are more likely to be achievable in experiments. Through the *in-silico* strain design case studies for producing succinate, 2,3-butanediol, and ethanol in yeast, we demonstrated that OptRAM can identify strategies that increase production beyond what is seen currently, or found as potential designs using alternative methods. We also validated the modified genes chosen by OptRAM in example cases against previous *in vivo* experiments in the LASER database. Additionally, we experimentally validated the ethanol strain design by evaluating its performance in fermentation. OptRAM provides a robust approach to strain design across gene regulatory network modification and metabolic engineering.

## Introduction

Microbial-based cell factories can be used to advance environmentally friendly and economically viable industrial bioprocesses. Various strategies have been suggested to modify industrial strains to improve desired product yields. Traditional methods of strain screening mainly rely on mating, hybridization and mutagenesis techniques [1,2], which are time consuming and costly, and have struggled to keep up with current industrial needs. In 1991, Jay Bailey proposed the term "metabolic engineering" to show how using recombinant DNA and other techniques could improve specific metabolic activity in cells by manipulating enzymes, transporters, and regulation to make cells meet human-specified goals [3]. Rational strain design methods suggest particular genes or enzymes to alter in order to achieve desired strain characteristics for metabolic engineering [4].

Systems biology is a powerful approach to uncover genotype-phenotype relationships, which can guide rational design-build-test iterations on strains to improve phenotypic properties in metabolic engineering. Next-Generation Sequencing (NGS) [5] and semi-automatic annotation techniques [6] have produced an increasing number of well annotated microbial genomes, enabling the collection of reasonably comprehensive information about which metabolic enzymes are encoded. This information has greatly contributed to the reconstruction of the genome-scale metabolic models of various organisms [7].

GEnome-scale metabolic Models (GEMs) are mathematical representations of the complete network of known biochemical reactions that can occur in a particular cell, assembled as a collection of metabolites, reaction stoichiometries, compartmentalizations, and gene-protein-reaction associations [8,9]. One of the main analysis approaches of GEMs is the well-known Flux Balance Analysis (FBA) [10], which can predict phenotypes for cells under different genetic and environmental conditions based on the stoichiometric matrix without requiring kinetic parameters [11,12]. It has been demonstrated that computational simulation on GEMs can predict effective engineering strategies for strain design [13,14]. Since the first strain design method OptKnock [15] was proposed in 2003, several computational methods for efficient automated identification of genetic strain modifications have been developed, such as RobustKnock [16], OptGene [17], OptORF [18], GDLS [19], and FSEOF (Flux Scanning based on Enforced Objective Flux) [20]. These algorithms have already yielded successful strain design applications. In an early example, Fong et al. designed *E*. *coli* strains for lactate production with a maximum 73% increase by using OptKnock [21]. Researchers from Tianjin University utilized a GEM of *B*. *subtilis* and elementary mode analysis to design an engineering strain for isobutanol production, and experimentally verified a 2.3-fold increase compared to wild type strain [22]. Recently, Otero et al. designed a strain using OptGene to overproduce succinate in *S*. *cerevisiae*, and experimentally validated a 43-fold improvement in succinate yield on biomass after directed evolutions [23].

However, a metabolic model alone has a significant limitation in revealing condition-specific metabolic activity [24,25] because gene regulation plays an important role in constraining the particular metabolism available under any given condition. Also, the complex crosstalking mechanisms between gene regulation and metabolism are not captured by a metabolic model alone. To overcome the limitation, methods that systematically integrate a transcriptional regulatory network and a metabolic network have been developed [26], including regulatory Flux Balance Analysis (rFBA) [27], steady-state rFBA (SR-FBA) [28], Probabilistic Regulation of Metabolism (PROM) [29], and Integrated Deduced REgulation And Metabolism (IDREAM), developed by our group [30]. Modification of gene regulatory circuits is an important strategy for transforming engineering strains [14]. In fact, modifications of regulatory factors (e.g. upregulation of biosynthetic pathway activators) contribute to more than half of the genetic operations in *E*. *coli* and *S*. *cerevisiae* engineering strains, but most of these interventions are based on human intuition [31]. Therefore, some strain design methods have utilized transcriptional regulation information to propose more effective metabolic engineering strategies. OptORF [18] was the first approach using integrated regulatory-metabolic models, which followed the framework of two-layer optimization as did OptKnock. In 2011, the heuristic strain design method OptGene also updated a version which introduced integrated regulatory-metabolic models [32]. In 2012, a series of approaches based on minimal cut sets (MCSs) was further developed to include a new tool (rcMCSs), that incorporates regulatory constraints [33]. However, since the above algorithms used manually curated integrated regulatory-metabolic models, where the regulatory network is a Boolean network, there are some limitations to application. Firstly, only some well-studied microorganisms may have existing integrated networks, such as *E*. *coli* [34], *M*. *tuberculosis*[35], and yeast [36]. Reconstructing such models requires extensive manual adjustment and additional information for generating Boolean logic rules in the regulatory network [37], which hinders the ability of these algorithms to be broadly applicable across many organisms. Secondly, these algorithms have to assume that the target gene is completely active or inactive, which ignores the range of possible regulatory intensities between regulatory factors and the target genes. In addition, Boolean networks can only suggest the manipulation of transcription factor by knockouts (ON to OFF) and cannot provide guidance for more quantitative adjustment of transcriptional regulation.

Recently, a method named Beneficial Regulator Targeting (BeReTa), used gene expression to infer the interaction between regulatory factors and target genes, combined with FSEOF [20] for identifying transcription regulators to enhance desired production. According to the correlations between the different transcription factor expression levels and target reaction flux rates, beneficial scores are calculated to judge whether the transcription factor can enhance or inhibit the target reaction. The algorithm was applied to *E*. *coli*, as well as *S*. *coelicolor*, which does not currently have an integrated metabolic-regulatory model. BeReTa represents a significant advance, but it cannot predict an expected product rate or yield of the mutant, or make predictions about the combined manipulations of multiple sites.

Herein, we report a new strain design algorithm, named OptRAM (**Opt**imization of **R**egulatory **A**nd **M**etabolic Networks), which can identify combinatorial optimization strategies including overexpression, knockdown or knockout of both metabolic genes and transcription factors, based on our previous IDREAM integrated network [30]. OptRAM also aims to achieve optimal coupling between biomass and target production, and can be used for strain design of bacteria, archaea or eukaryotes. The other advantage of OptRAM compared with previous heuristic approaches is that we systematically evaluated the implementation cost of different solutions and selected strain designs which are more likely to be achieved in experiments.

## Materials and methods

### Yeast metabolic network

*Saccharomyces cerevisiae* S288c has been studied and simulated extensively through a series of models [38] reconstructed based on the genome sequence and literature annotations [39]. We used the latest metabolic reconstruction, Yeast 7.6, which includes 3493 metabolic reactions, 2220 metabolites and 909 metabolic genes.

### Integrative regulatory-metabolic network modeling

Integration of a gene regulatory network with a metabolic network at the genome-scale poses significant challenges, in part because they are distinct network types requiring very different modeling frameworks. PROM uses probabilities to represent gene states and TF–gene interactions from abundant gene expression data, and then uses these probabilities to constrain the fluxes through the reactions controlled by the target genes [29]. A limitation of PROM is that a pre-built transcriptional regulatory network is required as an input. In our previous work, we developed a framework called Integrated Deduced REgulation And Metabolism (IDREAM), which uses bootstrapping-EGRIN-inferred [40,41] transcription factor (TF) regulation of enzyme-encoding genes, and then applies a PROM-like approach to apply regulatory constraints to the metabolic network. In Yeast, we collected 2929 microarray datasets with 5939 yeast genes and evaluated 392 of those genes as possible regulators. For each of the 5939 target genes, we constructed separate models from 200 randomly selected subsets of the 2929 experiments, as well as a 201st model constructed using the entire data set. This resulted in 201 generated gene regulatory models for each of the 5939 yeast genes, for a total of 1,193,739 models. For each gene, we estimated a false discovery rate (FDR) for each factor by tallying the fraction of models from random subsets that identified that factor as a regulator. Thus, if factor X was predicted to regulate gene Y in 191 of 200 models, then X would have an FDR = 1–191/200 = 0.045. If X is predicted to activate Y with an FDR of 0.045, only 4.5% of Y’s activity would be predicted to remain if X was deleted. If X is predicted to deactivate Y, then we use the much larger 1-FDR (e.g., 95.5% of activity) to represent that Y is somehow disturbed without a significant reduction in activity. We included only those interactions that passed an FDR cutoff of 0.05. Then we predicted whether a factor was an activator or repressor by testing if its mRNA expression was correlated or anti-correlated with the expression of its target using the model from the entire expression dataset. Finally, we retrieved an integrated regulatory-metabolic network including 2626 inferred influences consisting of 91 TFs transcriptionally regulating 803 genes encoding enzymes of the metabolic network.

It should be noted it is impossible to cleanly differentiate between the false discovery rate and the strength of the regulatory role for multi-cell microarrays or RNA-Seq because from bulk measurements we can’t differentiate a strong regulation occurring in a small portion of cells from a weak regulation happening in a large fraction of the cell population. In other words, because we are using a ground up mixture of cells, then we might reasonably expect a few cells with high expression related to a rarely used promoter to appear similarly to a low level, frequently used promoter. Perhaps single cell RNA-Seq will help disentangle this problem in the future.

### Strategies of gene expression mutation and the translation to reaction level

OptRAM is a meta-heuristic strain optimization method based on an integrated model of an inferred regulatory network and a constraint-based metabolic network. It aims to identify the modifications of TFs and metabolic genes, including overexpression, knockdown, and knockout, to achieve the maximal production of desired chemical. OptRAM will simulate a series of mutations to get the optimized strategy for target overproduction by simulated annealing. We adopt 11 kinds of mutations on gene expression of TFs or metabolic genes, represented as FC(TF) and FC(G), with overexpression and knockdown fold change of 2, 4, 8, 16, 32 respectively, and 1 knockout, as shown in Table 1.

**Table 1:** Mutations of the gene expression fold change (FC) over wildtype.

Overexpression FC	2	4	8	16	32
Knockout FC	0.001				
Knockdown FC	1/2	1/4	1/8	1/16	1/32

For knockout, the fold change is set as 0.001, because 0 has no logarithm, which will generate error for calculation of the target gene expression change.

The expression level of these genes will be translated to corresponding metabolic reactions by the integrative network. First, expression levels of metabolic genes are calculated according to expression of corresponding transcription factors. In the EGRIN algorithm, a linear equation of the target gene and the TFs is generated:

$$target={coeff}_{1}{TF}_{1}+{coeff}_{2}{TF}_{2}+\cdots {+coeff}_{n}{TF}_{n}$$ (1)

Where the variable *target* is the expression level of a target gene regulated by n TFs, *TF*_*i*_ are the expression level of these TFs, and *Coeff*_*i*_ are the corresponding coefficients of each TF.

In OptRAM, for a target gene regulated by one TF, *tfExpr* is the relative expression level of the mutated TF, then the relative expression level of the target gene is calculated as:

$$targExpr=2^{coeff\times \log_{2}\;tfExpr}$$ (2)

When a target gene is affected by more than one TF, the expression level of the target gene is calculated as:

$$targExpr=2^{\sum_{i}^{n}{coeff}_{i}\times \log_{2}\;{tfExpr}_{i}}$$ (3)

Having the relative expression level of all metabolic genes, the change of relevant reactions, represented as FC(R), is calculated according to the gene-reaction rules in the metabolic model. For reactions with ‘AND’ rules of different genes, we selected the minimum value of relative gene expression level, since ‘AND’ indicates that the combination of multiple enzymes is required, so the enzyme with lower expression determines the upper bound of the reaction rate. For reactions with ‘OR’ rules of different genes, the mean value of relative gene expression level is calculated, because ‘OR’ indicates that multiple enzymes have the same catalytic function and can be substituted for each other. Therefore, the average expression level of enzymes in the set can better reflect upper bound of the reaction rate. While the average was used herein, it would also be reasonable to use the max of the enzymes being expressed in this scenario.

### Flux constraints of reactions induced by the gene expression mutation

In order to simulate the flux change of reactions induced by the gene expression mutation, we first need a reference flux value for each reaction, which is obtained by pFBA (Parsimonious enzyme usage FBA) method [42]. pFBA is an algorithm based on FBA. For a metabolic network with M metabolites and N reactions, the FBA formulation is shown below:

$$\begin{array}{l} Maximize\;v_{objective}\\ Subject\;to\;\sum_{j=1}^{N}S_{ij}v_{j}=0,i=1,\ldots ,M\\ lb_{j}\leq v_{j}\leq ub_{j},j=1,\ldots ,N \end{array}$$ (4)

Where *S*_*ij*_ stands for the stoichiometric coefficient of metabolite *i* in reaction *j*, and *v*_*j*_ stands for the flux of reaction *j*, *lb*_*j*_ and *ub*_*j*_ are the constraints for reaction *j*. The most commonly used objective function (*v*_*objective*_) is biomass synthesis [43]. Here the simulation condition for yeast metabolic flux was set corresponding to the YPD medium, with glucose 20 g/L and blocking other carbon source.

The pFBA algorithm is divided into three steps. First, the max biomass rate is obtained by FBA with the original model. Secondly, the constraint of biomass is set equal to the max biomass value. Finally, a new objective function is set as the minimization of total flux values carried by all reactions, to generate the flux distribution. According to the reference flux values from pFBA and the level of expression change for mutated reactions, we set the new constraints of reactions as shown in Table 2, where FC(R) is the change of reaction, v is the reference flux value, *lb* and *ub* are the original lower bound and upper bound for the reaction.

**Table 2:** New constraints added to the mutated reactions induced by modified gene expression.

	v>0	v< = 0
FC(R)<1	[0, FC(R)*v]	[FC(R)*v, 0]
FC(R)>1	[FC(R)*v, ub]	[lb, FC(R)*v]

### New objective function in OptRAM

In the previous meta-heuristic strain optimization methods, such as OptGene, BPCY (biomass-product coupled yield) is used as the objective function [17]:

$$BPCY=\frac{Product\times Growth}{Substrate}$$ (5)

Where *Product* represents the flux of the reaction synthesizing the desired product, *Growth* represents the flux of biomass, and *Substrate* represents the uptake rate of the nutrient substrate. The ultimate goal of the optimization algorithm is to identify the mutated solution with the largest *BPCY* value, which ensures a considerable growth when improving the target product.

A limitation of the simulation using pFBA is that this framework does not guarantee that the target reaction flux will be coupled to biomass. That is, even if the *BPCY* score of a mutated solution is high, the flux value of the target reaction is unstable with the max biomass. Because the flux variability of target reaction is a wide range and the minimum flux may even be zero, there is no guarantee that the target product can have a certain output under natural growth. Moreover, since the objective function of pFBA is biomass, there is often no flux through the desired target reaction, although the flux range of that reaction may be 0 to a positive value. In this situation, *BPCY* remains 0 and the algorithm reports no feasible solution.

Therefore, we defined a new objective function (Eq 6) in OptRAM to couple maximizing biomass production and the target reaction of interest.

$$Obj=\frac{Target\times Growth}{Substrate}\times (1-\log\frac{Range}{Target})$$ (6)

Where $Target=\frac{V_{max}+V_{min}}{2},\;Range=\frac{V_{max}-V_{min}}{2},$ *V*_*max*_ is the maximum flux value of target reaction and *V*_*min*_ is the minimum flux value by FVA (flux variability analysis) [44]. *Target* means the average flux value of target product. *Range* is set to half of the interval between min and max target flux value. When *V*_*min*_ is 0, $\frac{Range}{Target}=1$, the coefficient $\left(1-\log\frac{Range}{Target}\right)$ is 1. And when *V*_*min*_ is greater than 0, $\frac{Range}{Target}>1$ the coefficient will be greater than 1, which is a reward coefficient for *BPCY*. Compared to BPCY, this objective function will induce solutions to have a higher and narrower flux range of target product, which reduces the uncertainty caused by alternative solutions in constraint-based modeling. Hence, by using the refined objective function, OptRAM can provide solutions with better biomass-product coupled.

### Implementation of strain optimization by simulated annealing

**Fig 1:**
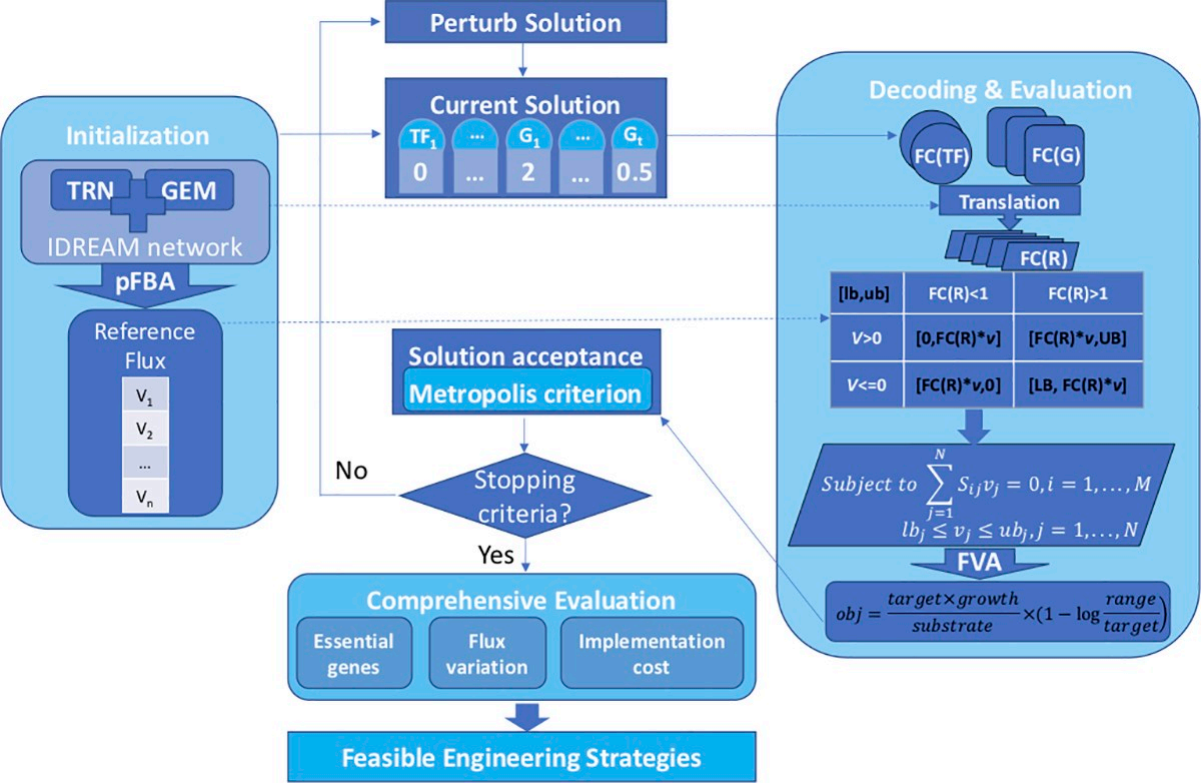
Overall framework of OptRAM. TRN indicates “Transcriptional Regulatory Network” and GEM indicates “GEnome-scale metabolic Model”. FC(TF), FC(G) and FC(R) indicate the fold of expression level of the transcription factors, metabolic genes and reactions respectively in the mutated strain over the wild type, which are set according to Table 1, ranging from 1/32 to 1/2 for knockdown in 5 levels, and 2 to 32 for overexpression in 5 levels. For knockout, the FC is set as 0.001 (0 will result in overflow). The formula of ‘obj’ indicates the objective function optimized by simulated annealing.

Fig 1 illustrates the strategy of the OptRAM approach, and the detailed pipeline can be downloaded from supplemental files (S1 Script and S1 Code). OptRAM requires a transcriptional regulatory network (or a gene expression data set) and a genome-scale metabolic model as input. Then IDREAM method will be run to get an integrated model. For organisms with no existing TRN, users can input a set of expression data from which the IDREAM method will automatically infer the TRN and generate an integrated model. Then the core strain design process within OptRAM will simulate a series of mutations to get the optimized strategy for target overproduction. The output from OptRAM includes the maximized objective score, flux of the target reaction, and the corresponding mutated solution with suggested modification of TFs and/or metabolic genes.

We used simulated annealing for the core strain design part, which is able to accept a worse solution in the early stage (avoiding getting stuck in local maximal), to pursue finding the global optimal screening for mutated models. The simulated annealing algorithm is derived from the simulation of the solid annealing process, an idea first proposed by Metropolis in 1953 [45]. In 1983, Kirkpatrick et al. introduced the idea of simulated annealing algorithm into the field of optimization problems, making the algorithm practical in engineering [46]. The simulated annealing algorithm introduces Metropolis criterion, which help escaping local optimum, to accept new solutions, including not only better solutions but also worse ones, according to the probability. By simulating the drop of temperature, the algorithm controls parameters during the process and gives an approximate optimal solution in polynomial time.

In our algorithm, we replace the internal energy of the annealing process with the refined objective function *Obj* (Eq 6), which is the prospective score for each mutated strain. The following steps show the implementation of the simulated annealing algorithm in our specific optimization problem:

1. Initialize the simulated annealing parameters, including the initial temperature *T*_*0*_ of the control parameter *T*, the attenuation factor (*α*<1), and the maximum number of iterations *L* at each temperature. Then generate the initial solution, *Ind*_*0*_.

2. When *T = T (k)*, search *L* times according to the following process:

(1) For the current solution *Ind*_*k*_, randomly mutate the expression of TFs and metabolic genes, translate to effects on reaction flux, and get a new *Obj* score and a new mutation solution *Ind'*_*k*_.

(2) Calculate *ΔObj = Obj(Ind'*_*k*_*)—Obj(Ind*_*k*_*)*, where *Obj(Ind)* is the value of objective function for each solution.

(3) If *ΔObj > 0* then *Ind'*_*k*_ is received as the new solution, let *Ind*_*k*_ *= Ind'*_*k*_; otherwise generate a random number *R* on the even distribution in (0,1), calculate the probability *P* according to Metropolis criterion:

$$P=e^{\frac{\Delta Obj}{T(k)}}$$ (7)

If *R<P*, then accept the new solution, let *Ind*_*k*_ *= Ind'*_*k*_; otherwise keep the current solution;

(4) If the number of iterations is less than *L* at this temperature, repeat step 2; otherwise, proceed to step 3.

3. If the convergence condition is satisfied, the algorithm ends and the current solution is the optimal solution, represented by a two-dimensional array of the mutated strain. The first array contains all the mutated gene IDs. The second array stores the expected fold change of expression level of the mutated gene over the wild type expression. The convergence condition is that the value of objective function has not improved for a number of continuous temperatures (here we set 100 temperatures); otherwise, proceed to step 4.

4. Decrease the control parameter *T*, let *T (k + 1) = T (k) ×α*, simultaneously reset the number of iterations and go to step 2.

### Comprehensive evaluation of solutions

#### Post-processing of the optimized solution

First, we eliminate redundancies in the optimized solution. For each candidate site in the optimal solution, we set gene expression to the original level of wild type, then recalculate the objective function to compare with the objective score of the optimal solution. If the score does not decrease, this mutation site will be excluded. Second, we check critical metabolic genes and reactions. Since a transcription factor usually affects more than one target gene, and one metabolic gene may catalyze more than one reaction, we need to search for critical metabolic genes and reactions for further analysis. We list all changed metabolic genes/reactions involved in the optimal solution and then examine their effects by excluding each mutated gene/reaction individually. We calculate the objective score of new models and compare it to the original score. The genes/reactions with the ratio between new and original score less than 0.9 are defined as critical genes/reactions.

#### Exclude solutions with essential genes knocked out or knocked down

We collected a list of essential genes from SGD (Saccharomyces Genome Database) [47] and DEG (Database of Essential Genes) [48], in supplemental S1 Table. There are 1192 and 1110 essential genes annotated in SGD and DEG respectively, among them 1021 essential genes are shared and 110 of them mapped to the Yeast metabolic network. In OptRAM, the solutions with essential genes knocked out or knocked down will be excluded, and the TF modification inhibiting essential genes will also be excluded, because such alterations might cause no growth of cell.

#### Implementation cost analysis based on network path

Usually strategies such as simulated annealing will find several different solutions with equivalent (or nearly equivalent) predicted optimal performance strain designs. Therefore, we implemented a metric to compare the implementation cost of different strategies in order to select designs that are easier to experimentally implement. To elucidate how the strategy of optimal solution can achieve the yield of the target product, we analyzed the main path from substrate to target using a directed network with reactions as nodes and metabolites as edges. Here, we excluded the “minor” metabolites (i.e. highly abundant in the network), including *H*^*+*^, *H*_*2*_*O*, *O*_*2*_, *CO*_*2*_, *ATP*, *ADP*, *AMP*, *dATP*, *dADP*, *dAMP*, *NAD*, *NADH*, *NADP*, *NADPH*, bicarbonate, phosphate, 5-phosphoribosyl-ATP, 3,5-cyclic AMP. The direction is determined by the flux value of pFBA with the mutated model. When searching for the main path from nutrient substrate to target product, we do not simply identify the shortest path connecting these two exchange reactions, because many reactions on the shortest path may carry zero flux. The shortest path may not reflect the actual flow of substance. We instead searched for the path that maximizes the minimum flux value of the entire path, which can reflect the actual substance flow from carbon substrate to target product to a certain extent.

Next, we constructed another undirected graph to analyze the relationship of critical reactions to the main path. If we were to use a directed graph, we would not be able to observe the connection of the main path with critical reactions having no flux. We then identify the shortest paths between these critical reactions and the main path as branch paths. For a mutant strain, we calculate a score according to connection of branch paths to the main path:

$$Score=\frac{\frac{\sum_{1}^{k}dist[i][j]}{C_{k}^{2}}\times \sum Branch\_Length}{Max\{Degree[i]\}}$$ (8)

Where the numerator is the average distance between all junctions of branch paths and the main path ($\frac{\sum_{1}^{k}dist[i][j]}{C_{k}^{2}}$) multiplied by the total length of all branch paths (∑*Branch*_*Length*), and the denominator is the largest numbers of branches that connected to one node on the main path (*Max*{*Degree*[*i*]}). A smaller score means a higher concentrated level of the engineered branches, which corresponds to a lower cost implementation.

**Fig 2:**
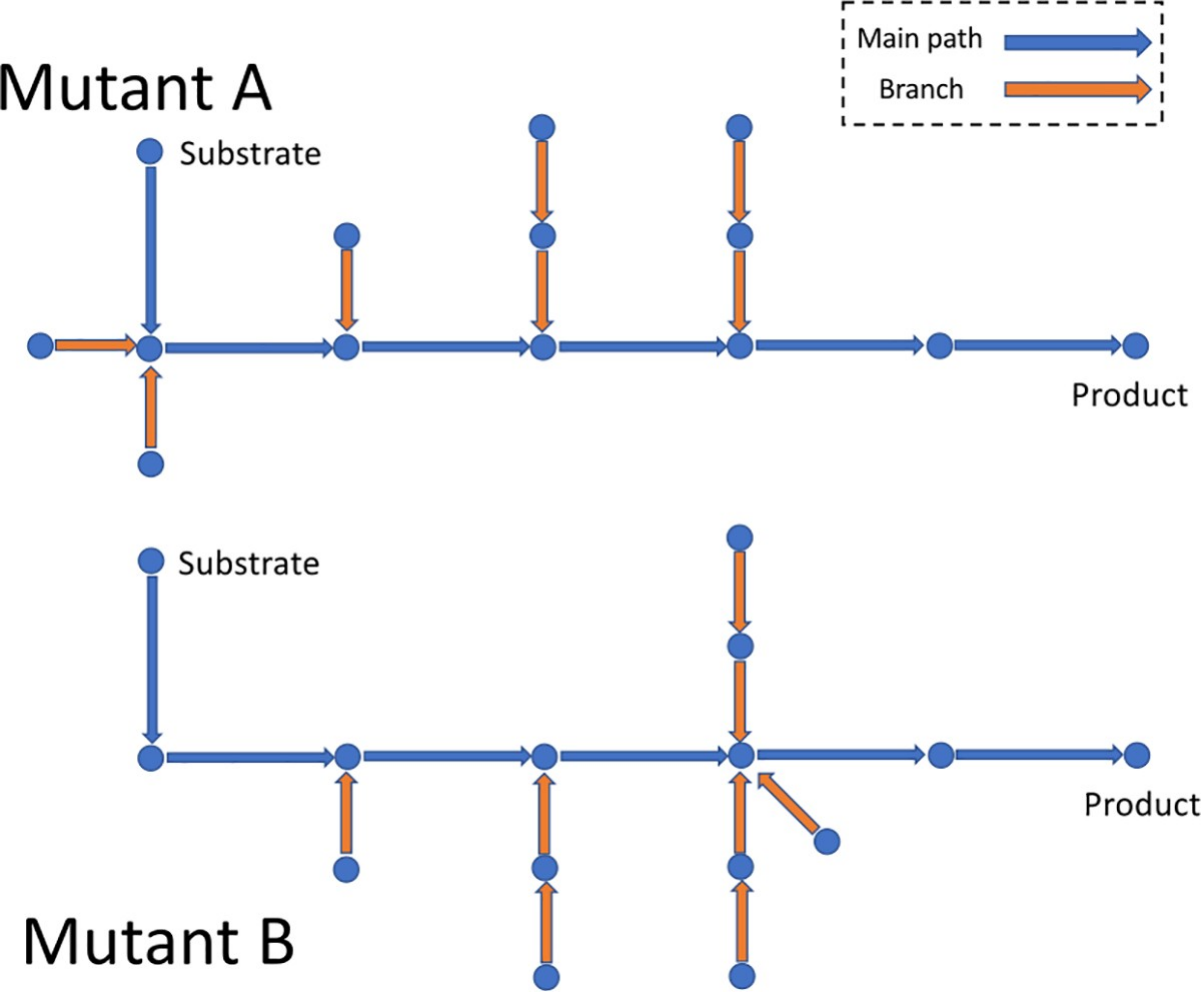
A diagram of implementation cost calculation according to connection of branch paths to the main path. This figure shows the main path and branches of two hypothetical mutant strains. Blue circles indicate metabolites, arrows in blue and orange indicate reactions on the main path and branch paths respectively.

As shown in Fig 2, mutant A and mutant B are two hypothetical mutants for one desired product. For mutant A, the average distance between the 4 junctions of branch paths and the main path is (1+1+1+2+2+3)/6 = 1.67. The total length of all branch paths is 1+1+1+2+2 = 7. The largest number of branches that connected to one node on the main path is 2. Then the implementation cost score of mutant A is 1.67*7/2 = 5.83. For mutant B, the average distance between the 3 junctions is (1+1+2)/3 = 1.33. The total length of all branch paths is 1+1+2+2+2 = 8, and the largest number of branches connected on the main path is 3. Then the implementation cost score of mutant B is 1.33*8/3 = 3.55. Mutant A has a higher score than mutant B, which means engineered branches in mutant A are lowly centralized. If these two mutants have very close objective scores, mutant B will be the prioritized choice.

#### Measure the overall adjustment between modified strain and the wild type

In OptRAM, another factor affecting the choice from multiple optimal solutions is the overall adjustment between mutant strains and the wild type. We prefer to select parsimonious solutions requiring fewer genetic interventions to ensure the robustness of biological system in case of unexpected impact and to make the strain design experiments more feasible. To quantify the extent of genetic adjustment, we calculated the Cosine function of the two vectors including all reaction flux values for particular mutant strain and the wild type.

$$Cosine=\frac{\left|\vec{v_{mt}}∙\vec{v_{wt}}\right|}{\left|\vec{v_{mt}}||\vec{v_{wt}}\right|}$$ (9)

Where *v*_*mt*_ is the vector of flux values of all reactions in mutated model and *v*_*wt*_ is the flux vector for wild type. The range of Cosine is between -1 and 1, with the larger value means smaller variation of the mutated strain.

#### Strain and fermentation

The *S*. *cerevisiae* strain used for the genes disruption is laboratory strain S288C, which was cultured in the YPD medium to the mid-log phase (around 18 h) in shake flasks and inoculated into the fermenter with 1 L working volume. A 10% inoculum was provided, and culturing was performed at 30°C, pH 4.5, 150 r/min, without aeration. The *E*. *coli* DH5α was used for construction of gRNA expression vector, and was routinely grown on LB medium at 37°C. YPD medium (g/L): 20 glucose, 10 yeast extract, 20 peptone; YPD plates: YPD medium and agar 15 g/L. LB medium (g/L): 5 yeast extract, 10 tryptone, 10 NaCl; LB plates: LB medium and agar 15 g/L. Yeast fermentation medium (g/L): 90 glucose, 10 yeast extract, and 20 peptone. Selection plates containing corresponding antibiotics were used for selecting transformants. All experiments were triplicates.

#### Target product and biomass measurement

A 2-mL of the sample was collected from the fermentation broth at 24 h. The cells were removed by centrifugation (10000 × g for 5 min), and the supernatant was subjected to glucose, ethanol quantification using HPLC (Model: Waters 1525) with an RI detector (Model: Waters 2414) at 50 ºC. An ion-exclusion column (Model: Aminex HPX-87 H 300 × 7.8 mm, Bio-Rad, USA) was employed to separate metabolites. While 10 mmol/L H_2_SO_4_ served as mobile phase at the flow rate of 0.6 mL/min. The cell growth was measured using the optical density at 600 nm.

#### Mutated strain construction

*BDH1* knock-out strain was constructed through homologous recombination of a resistance gene Kan MX4, which was amplified from HO plasmid using primer 1 and 2 containing homologous sequence of *BDH1*. The PCR products were purified and transformed into S288c. S288cΔ*BDH1* were selected by YPD with G418.

*MDH2* and *COX4* knock-out strain were constructed by CRISPR/Cas9 as Zhang et. al described [49]. First, gRNA-expressing plasmid was constructed by introducing synthesized *MDH2* or *COX4* gRNA DNA fragments into the *Bsa*Ⅰ digested pRS42H-gRNA. Second, double-stranded 90 bp repair DNA for *MDH2* or *COX4* disruption was amplified by introducing stop codon TAA into the PAM sequence, respectively. Third, Cas9-NAT was transformed into cells to generate Cas9 protein. Lastly, pRS42H-gRNA-*MDH2* or pRS42H-gRNA-*COX4* were transformed together with the 90 bp donor DNA (~2 μg) into Cas9-expressing strain and selected by nourseothricin and hygromycin, respectively.

## Results

### Integrated regulatory-metabolic network of yeast

We used flux variability analysis on the Yeast7.6 model to analyze the range of possible flux values for all reactions and excluded the reactions that could not have a non-zero value since these would not have any effect on the strain optimization calculations. The processed metabolic model has fewer reactions, metabolites and genes compared to the original Yeast7.6 model (Table 3). By using the IDREAM approach, we integrated the deduced regulatory network from 2929 microarray datasets with the Yeast7.6 metabolic network to generate an integrative gene regulatory-metabolic network (Table 3).

**Table 3:** Components in the integrated regulatory-metabolic network of yeast.

**Metabolic network**	**Reactions**	**Metabolites**	**Genes**
Yeast7.6	3493	2220	909
Processed	2676	1629	714
**Regulatory network**	**TFs**	**Targets**	**Regulations**
Raw	396	5938	208487
FDR< = 0.05	91	628	2146

Processed metabolic network means the reactions with zero flux are excluded. Regulatory network incorporated with metabolism includes the interactions filtered by FDR < = 0.05.

### Case study: Strain optimization for succinate production

We performed 10 parallel simulated annealing runs, which set succinate as the target product, and obtained 10 solutions (Supplemental S3 Table). We first filtered the solutions with *in silico* knockdown or knockout of known essential genes. Then we selected solutions with the maximal fluxes of the target reaction. For solutions with the same predicted yield in succinate, we chose the one with a smaller implementation cost and a larger Cosine function representing smaller variation of genetic manipulation. Finally, we selected one optimized strain design from ten runs, with the mutation sites and expression modifications shown in Table 4.

**Table 4:** Modification sites in best solution for succinate as target.

Gene	*THI3*	*CYS4*	*ACO1*	***PDR1***	***PHO4***
Overexpression fold change	32	32	4	4	4
Gene	*CTP1*	*MDH3*	*PDC5*	*COX7*	*SER1*
knockdown fold change	1/2	1/8	1/2	1/4	0

**Fig 3:**
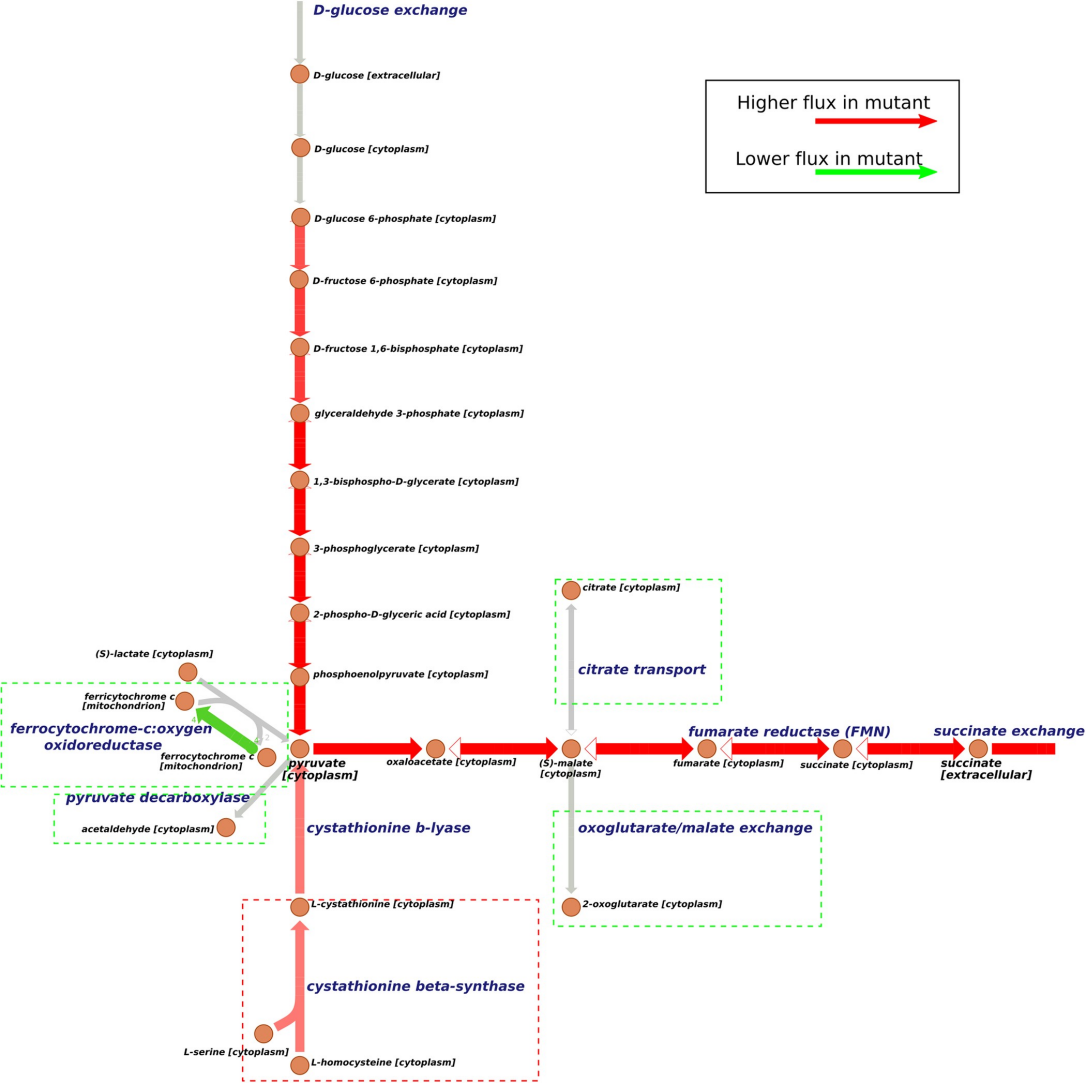
Flux comparison of a mutated model and wild type for succinate overproduction. This figure shows the main path of succinate production in yeast and critical reactions identified by OptRAM. Solid arrows indicate the direction of metabolic reactions. Red arrows indicate that the fluxes are predicted to be higher in the mutated strain and green arrows indicate the flux is predicted to be lower than in wildtype. Gray arrows indicate the reactions are not significantly different between the designed strain and the wildtype. Red dotted boxes highlight the critical up-regulated reactions and green ones highlight the down-regulated reactions.

The upper two rows show mutation sites with overexpression manipulation, while the rows below show mutation sites with knockdown manipulation. Corresponding numbers are perturbation fold change of expression as defined in Table 1. *PDR1* and *PHO4* are TFs, and others are metabolic genes. For this complicated combination of TFs and metabolic genes, we analyzed the critical sites corresponding to metabolic reactions. A ‘critical reaction’ here means that the removal of the reaction can cause more than a 10% reduction in the objective function (See Methods). There are 5 critical reactions (marked in Fig 3) involved in this solution: up-regulation in cystathionine beta-synthase, down-regulation in ferrocytochrome-c: oxygen oxidoreductase, pyruvate decarboxylase, citrate transport, and oxoglutarate/malate exchange.

There are two main processes in the production of succinate from glucose (Fig 3). First, glucose is used to make pyruvate in cytoplasm. Next, pyruvate converts to oxaloacetate, then to malate, then to fumarate, and finally to succinate. Comparison of the flux values in the two models revealed that the succinate exchange flux increases about 66-fold theoretically in the strain-optimized model. All critical reactions we found are closer to the path from pyruvate to succinate. Up-regulation in cystathionine beta-synthase will promote more L-cystathionine to pyruvate. Decreasing the constraints of ferrocytochrome-c:oxygen oxidoreductase and pyruvate decarboxylase directly affects the pyruvate flux from lactate and acetaldehyde. So pyruvate mostly comes from phosphoenolpyruvate in the mutated strain. Meanwhile, citrate transport and oxoglutarate/malate exchange are limited to prevent malate in cytoplasm to transport to mitochondrion, which enable more malate flux to succinate synthesis. We compared the best solution in Table 4 with the previous literature in which the succinate was successfully improved in yeast assisted by strain design method [23]. This study used OptGene to get the optimized strategy including deletion of *SDH3*, *SER3* and *SER33*. *SDH3* (Succinate dehydrogenase) catalyzes the reaction from succinate to fumaric acid, its deletion will obviously promote flux to succinate. Deletion of *SER3* and *SER33* can facilitate TCA Cycle, and improve succinate. We also identified SER1 as engineering site by OptRAM. To compare with other strain design methods, we chose the widely used OptFlux [50] to find overexpression, knockdown, and knockout of metabolic genes. For the mutant models generated from literature, OptFlux, and OptRAM, we used FVA to investigate the range of target reaction under constraints of being able to achieve 99% and 50% biomass respectively. The 99% biomass constraint means that the lower bound of biomass reaction is set as the 99% of maximum biomass in each mutant model respectively, similarly for 50% biomass. The 99% biomass is a relatively strict constraint on biomass and the 50% biomass is a loose constraint. A strict constraint on biomass will make the flux range of target reaction much more narrow. We compared the resulting flux range of these mutant models with the wild type model (Table 5).

**Table 5:** Comparison of flux ranges of succinate production in mutant models by different methods and literature.

	Succinate (99%biomass)	Succinate (50%biomass)	Biomass
Wild type	[0.00, 0.18]	[0.00, 8.85]	0.95
Literature^a^	[0.10, 0.43]	[0.00, 8.20]	0.90
OptFlux	[6.78, 7.46]	[0.03, 11.91]	0.51
OptRAM	[11.62, 11.88]	[1.11, 13.20]	0.26

Values in the square brackets are minimal and maximal flux values of succinate exchange reaction respectively. The flux unit is mmol/KgDW.h.

^a^Strain design from literature with experimentally validation[23].

When biomass is constrained to 99% of the maximal theoretical value, our strategy makes an obvious improvement for predicted succinate production, even the minimal succinate exchange flux is higher than the max value in wild type and the strategy in literature [23]. Although the max biomass flux is relatively low, it is still acceptable for strain growth. When biomass is constrained to half of the theoretical max value, the model comparison suggests that our strategy makes succinate production strongly coupled with growth. The performance of solution from OptFlux seems good, but the overall interval in OptRAM solution is much larger than other strategies.

We also compared all the mutation sites from 10 solutions with the experimental design in LASER database, as shown in S4 Table. There are 9 genes presenting in more than 2 solutions matched with the experimental modifications in LASER, such as *MDH2*, *SDH3*, and several mutated TFs, including *CAT8*, *HAP2*, have targets modification improving succinate validated in LASER. Overexpression of *CAT8* can promote succinate production by increasing flux of glycolysis and TCA Cycle, *DIC1* and *ICL1* are regulated by *CAT8*, which have significant effects on succinate reported by Agren *et al* [51] and Otero *et al* [23]. *HAP2* is a global regulator responsible for respiration gene expression, one of its targets *IDP1* has been validated to improve succinate [52].

### Case study: Strain optimization for 2,3-Butanediol production

**Fig 4:**
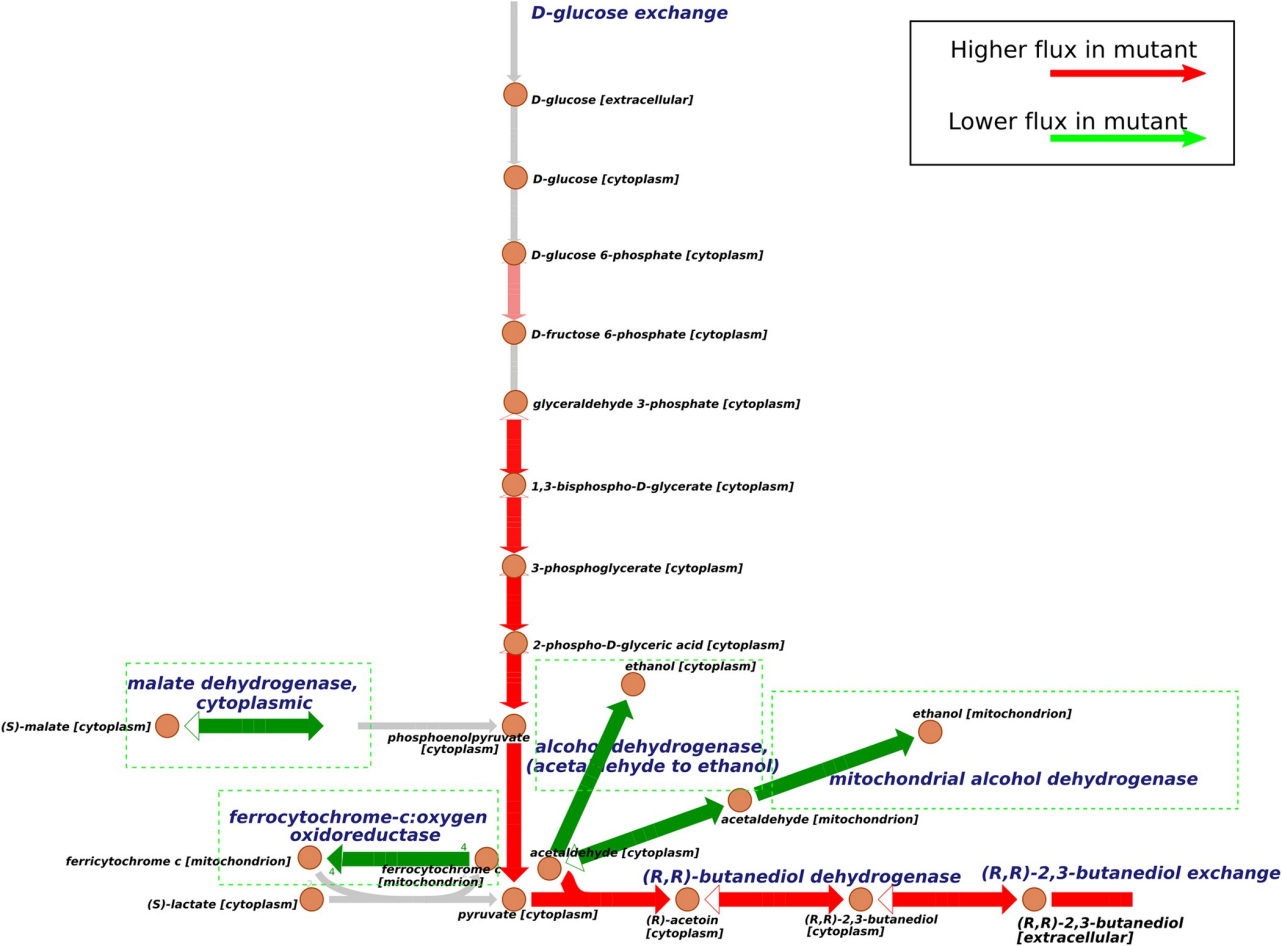
Flux comparisons of mutated model and wild type for 2,3-butanediol overproduction. This figure shows the main path of 2,3-butanediol production in yeast and effective reactions identified by OptRAM. Solid arrows indicate the direction of metabolic reactions. Red arrows indicate that the fluxes are predicted to be higher in the mutated strain and green arrows indicate the flux is predicted to be lower than in wildtype. Gray arrows indicate the reactions are not significantly different between the designed strain and the wildtype. Green dotted boxes highlight the critical down-regulated reactions.

We performed 10 parallel simulated annealing runs with 2,3-butanediol as target product (Supplemental S3 Table). According to the filtering process similar with succinate case, we selected one optimized solution from the results of the ten runs. Table 6 shows the mutation sites suggested by OptRAM, among which *GLN3* and *RTG3* are TFs. There are 4 critical metabolic reactions (marked in Fig 4) involved in this mutated model, respectively catalyzed by mitochondrial alcohol dehydrogenase, ferrocytochrome-c:oxygen oxidoreductase, malate dehydrogenase, and cytoplasmic alcohol dehydrogenase (acetaldehyde to ethanol), all of them are predicted to be knocked down.

**Table 6:** Manipulation sites in best solution for 2,3-butanediol as target.

Gene	*SOL3*	***GLN3***		
Overexpression fold change	8	8		
Gene	*COX4*	*POX1*	*MDH2*	***RTG3***
knockdown fold change	1/4	1/4	1/4	1/32

There are two main processes in the production from glucose to 2,3-butanediol (Fig 4), just as for succinate. Pyruvate comes from glucose and is converted to acetoin and then to 2,3-butanediol. By comparing the flux values in the two models (mutated and wild type), the flux of 2,3-butanediol exchange reaction increases about 61-fold theoretically in the mutated model. Reduction of ferrocytochrome-c:oxygen oxidoreductase will affect pyruvate in cytoplasm coming from lactate. Repressing alcohol dehydrogenase and mitochondrial alcohol dehydrogenase, both of which convert acetaldehyde to ethanol, will prevent acetaldehyde to ethanol. Downregulation of malate dehydrogenase changes the direction of the reversible reaction from malate to more oxoglutarate, and promote the flux of pyruvate to final 2,3-butanediol.

We also compared the best solution in Table 6 with the previous literature in which Ng *et al* succeeded in improving 2,3-butanediol in yeast [53]. They used OptKnock to explore optimization design including the deletion of *ADH1*, *ADH3* and *ADH5* under an anaerobic condition. We used OptRAM to identify knockdown of *ADH3*, and the TF *STE12* regulating *ADH1* and *ADH3* to improve 2,3-butanediol. Also, we ran OptFlux to get strain design solution for comparison. For the mutant models generated from literature, OptFlux, and OptRAM, we used FVA to obtain the range of 2,3-butanediol target under constraints of being able to achieve 99% and 50% biomass, respectively. The comparison of mutant models with wild type model is shown in Table 7.

**Table 7:** Comparison of flux ranges of 2,3-butanediol production in mutant models by different methods and literature.

	2,3-butanediol (99%biomass)	2,3-butanediol (50%biomass)	Biomass
Wild type	[0.00, 0.12]	[0.00, 5.88]	0.95
Literature^b^	[5.07, 6.42]	[0.00, 9.76]	0.24
OptFlux	[0.00, 5.90]	[0.00, 8.34]	0.47
OptRAM	[6.86, 7.20]	[0.00, 8.54]	0.37

Values in the square brackets are minimal and maximal flux values of 2,3-butanediol exchange reaction respectively. The flux unit is mmol/KgDW·h.

^b^Strain design from literature with experimentally validation[53].

Similarly, when biomass is constrained to 99% of the max theoretical value, even the minimal flux value of 2,3-butanediol exchange reaction by OptRAM is higher than other strategies. While the minimum predicted target flux in OptFlux drops to 0 with the constraints of 99% max biomass, it means the good performance of OptFlux for succinate production coupled with biomass might be a case-specific outcome, whereas OptRAM can ensure the coupling for overproduction of different targets, because of the improved objective function.

We also compared all the mutation sites from 10 solutions with the experimental design in LASER database (Supplemental S4 Table). There are 6 genes presenting in more than 2 solutions matched with the experimental modifications in LASER, such as *ADH3*, *GPD2*. Knockdown of *ADH3* (alcohol dehydrogenase) can keep more flux to acetaldehyde and impress the flux to ethanol. Deletion of *GPD1/2*(glycerol-3-phosphate dehydrogenase) can improving ethanol by effectively decreasing flux to glycerol [54]. We also found several mutated TFs, such as *STE12* and OAF1, have targets modification improving 2,3-butanediol validated in LASER. *STE12* is an important global regulator for yeast growth, whose target genes including *ADH1/2*, *ALD6*, *BDH1*, *GPD1/2*, have been reported as effective modification for improving 2,3-butanediol [49]. *OAF1* also regulates *ADH1/2*. It demonstrated that by our strain design method based on integrated model, the global TF could be identified for modification to accomplish the roles of several metabolic genes.

### Case study: Strain optimization for ethanol production

**Fig 5:**
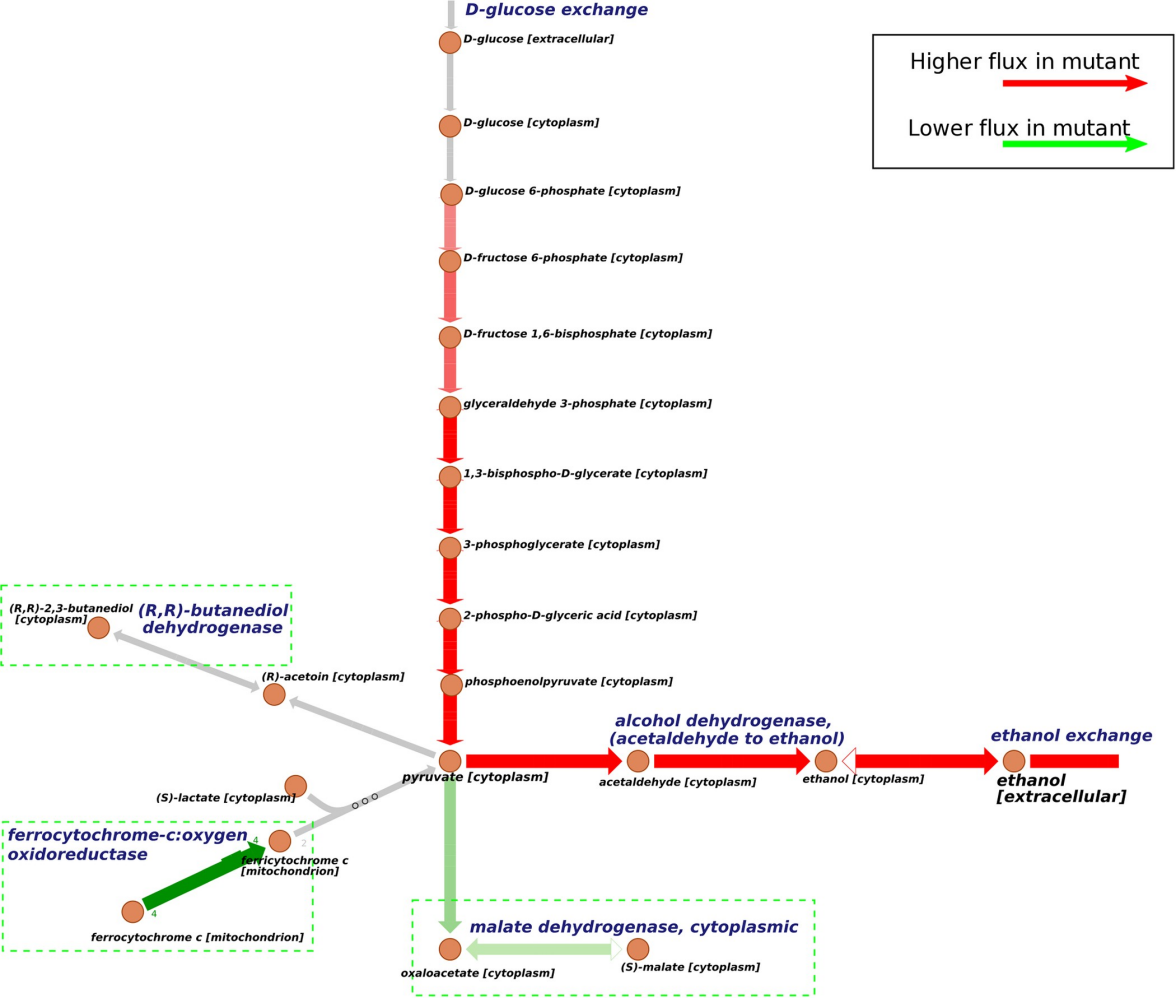
Flux comparisons of mutated model and wild type for ethanol overproduction. This figure shows the main path of ethanol production in yeast and critical reactions identified by OptRAM. Solid arrows indicate the direction of metabolic reactions. Red arrows indicate that the fluxes are predicted to be higher in the mutated strain and green arrows indicate the flux is predicted to be lower than in wildtype. Gray arrows indicate the reactions are not significantly different between the designed strain and the wildtype. Green dotted boxes highlight the critical down-regulated reactions.

We performed 10 parallel simulated annealing runs with ethanol as target product (Supplemental S3 Table). According to the similar filtering process, we selected one optimized solution from the results of the ten runs. Table 8 shows the mutation sites suggested by OptRAM. There are 3 critical metabolic reactions (marked in Fig 5) involved in this mutated model, respectively catalyzed by (R,R)-butanediol dehydrogenase, ferrocytochrome-c:oxygen oxidoreductase and malate dehydrogenase in cytoplasm, all of them are predicted to be knocked down.

**Table 8:** Manipulation sites in best solution for ethanol as target.

Gene	*DGA1*	***HAP2***		
Overexpression fold change	8	4		
Gene	*COX4*	*BDH1*	*ELO3*	*MDH2*
knockdown fold change	1/16	1/2	1/4	1/4

**Fig 6:**
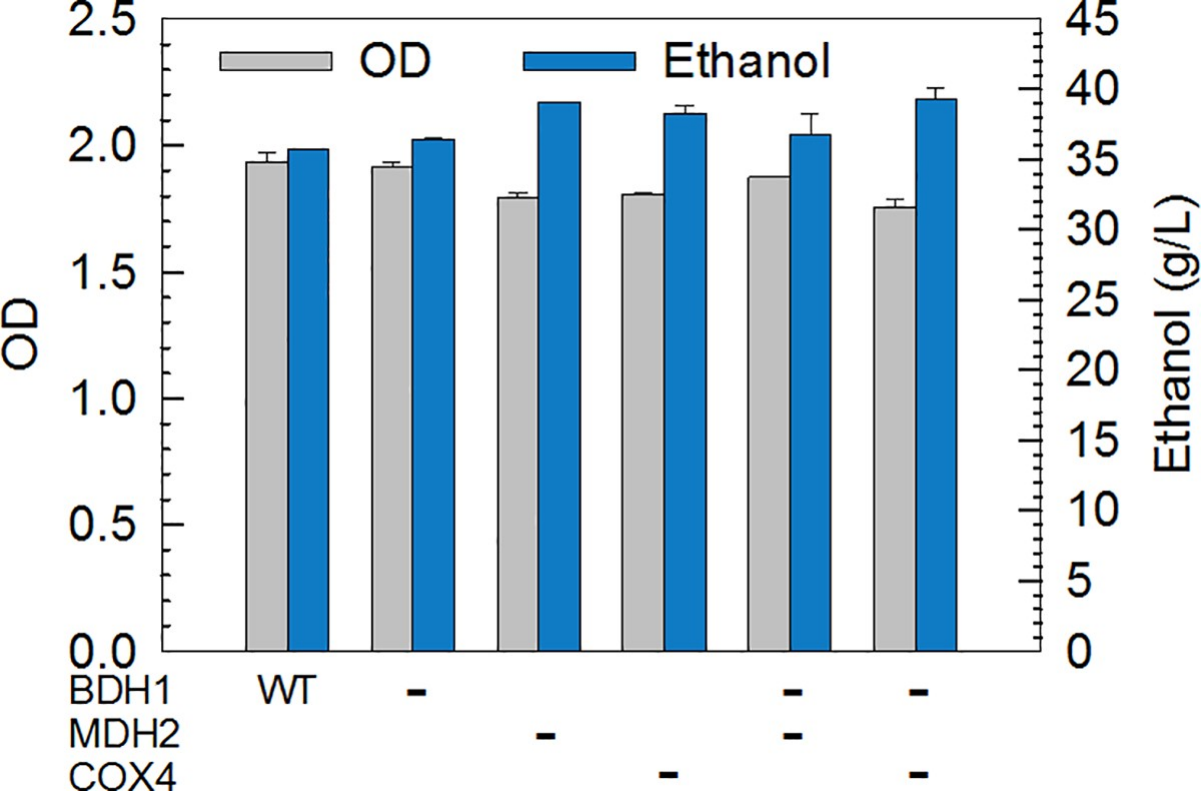
The cell growth and ethanol production of gene-deleted strains. Yeast *Saccharomyces cerevisiae S288C* was incubated in YPD medium consisting of glucose 90 g/L, yeast extract 10 g/L, and peptone 20 g/L. Samples were taken at 24 h. All experiments were triplicates.

We experimentally implemented the OptRAM-based design by modifying yeast strains using CRISPR-Cas9 and measuring the effects on ethanol production by fermentation. Three genes (*BDH1*, *MDH2* and *COX4*) in totally different pathways were firstly considered simultaneously to enhance the ethanol pathway, which were deleted separately as well as in combination. The growth of the modified strains was slightly reduced compared to wildtype with all of the strains showing an increase in ethanol production (Fig 6). The performance of these genes depends on their molecular function in yeast cells. *BDH1* encoding 2,3-butanediol dehydrogenase had the smallest effect on growth and ethanol production. *MDH2* and *COX4* encode cytoplasmic malate dehydrogenase in the TCA pathway and subunit IV of cytochrome c oxidase in mitochondrial inner membrane electron transport chain, respectively. These two pathways are primary competitors to the fermentation pathway for carbon flux. Thus the significant ramping up of ethanol, as well as growth decline, was observed on the yeast cells without *MDH2* or *COX4*. Since the metabolic burden and metabolic optimization are two sides of coin, the fermentation results of double deletion become much more complex. The yeast without genes *BDH1* and *COX4* produced the highest ethanol, but the yeast without *BDH1* and *MDH2* even could not keep consistent ethanol titer. Taken together, the prediction of OptRAM for three key genes to improve ethanol fermentation has been partially validated. However, the metabolic burden must be taken into consideration especially when carrying out multiple gene manipulation on microbes.

We also compared the best solution with the previous literature in improving ethanol in yeast [55]. The best strategy in this study included the deletion of *GDH1* and overexpression of *GLT1* and *GLN1*. Also, we ran the optimization algorithm OptFlux to get its strain design solution. For the mutant models generated from literature, OptFlux, and OptRAM, we used FVA to obtain the range of ethanol target under constraints of being able to achieve 99% and 50% biomass, respectively (Table 9).

**Table 9:** Comparison of flux ranges of ethanol production in mutant models by different methods and literature.

	ethanol (99%biomass)	ethanol (50%biomass)	Biomass
Wildtype	[0.00, 0.21]	[0.00, 10.93]	0.95
Literature^b^	[0.00, 0.22]	[0.00, 10.94]	0.94
OptFlux	[8.13, 12.88]	[0.00, 17.20]	0.39
OptRAM	[15.70, 15.98]	[4.78, 18.16]	0.26

Values in the square brackets are minimal and maximal flux values of ethanol exchange reaction respectively. The flux unit is mmol/KgDW·h.

^b^Strain design from literature with experimentally validation [55].

When biomass is constrained to 99% of the max theoretical value, even the minimal flux value of ethanol exchange reaction in OptRAM is higher than other strategies. When biomass is constrained to half of the theoretical max value, we can still see our strategy make ethanol production coupled with growth.

In addition, we compared all the mutation sites from 10 OptRAM solutions with the experimental design in the LASER database (Supplemental S4 Table). *MDH2* (malate dehydrogenase) is the top recurrent mutation found by all 10 solutions, whose deletion represses the TCA Cycle, increases flux of anaerobic respiration, and improves ethanol production [56]. *BDH2* is another recurrent mutation found by 8 solutions, whose knockdown can increase flux from acetaldehyde to ethanol. There are also some TFs having targets whose modification improves ethanol validated in LASER. HCM1 is found as a TF to modulate using OptRAM and it regulates *ACC1*, *GLN1*, and *TKL1*, all of which have been reported to improve ethanol production as reported in LASER [57–59]. For example, knockdown of *HCM1* can repress *ACC1* (acetyl-CoA carboxylase), and make more flux from pyruvate to ethanol.

### Comparison of OptRAM with other strain design methods

We systematically compared our OptRAM method with existing representative constraint-based strain optimization methods. OptKnock is the first method in this field, OptGene is the first trial of meta-heuristic method, OptORF is the first strain design method utilizing regulatory information, and BeReTa is the latest method utilizing regulatory information, which can suggest manipulations of transcription factors to be knocked down or overexpressed. Table 10 shows the different properties of these methods.

**Table 10:** Comparison of characteristics of some computational strain design methods.

	OptKnock	OptGene	OptORF	BeReTa	OptRAM
Type of algorithm	Bilevel	Meta-heuristic	Bilevel	Flux distribution	Meta-heuristic
Integration of TRN	No	No	Boolean	Inferred	Inferred
Metabolic gene strategies	Knockout/down	Knockout/down & Overexpression	Knockout/down & Overexpression	/	Knockout/down & Overexpression
Transcription factor strategies	No	No	Knockdown	Knockdown& Overexpression	Knockout/down& Overexpression
Coupled with Biomass	Maximal target	Maximal target	Maximal target	Maximal target	Ensure coupling & Maximal target
Mutant sites combination	Yes	Yes	Yes	No	Yes
Max yield prediction	Yes	Yes	Yes	No	Yes
Comprehensive evaluation of solutions	No	No	No	No	Essential genes; Implement cost; Global adjustment

We can see that only OptORF, BeReTa and OptRAM integrate a regulatory network. OptORF utilizes the Boolean network which has more limitations in practice, while BeReTa cannot give a combination of multiple engineering sites. OptRAM can simultaneously identify transcription factors and metabolic genes to be targeted for overexpression, knockdown, and knockout. Solutions from OptRAM ensure that the target product is better coupled with cell growth, and further systematical evaluation can help biologists to choose a relatively reliable solution for experiment validation.

When performing OptRAM on MATLAB (2017a) with GUROBI version of 7.5, the average time of one SA process is 3.7 hours. Overall, the computation time is comparable to the time running optimization algorithm once on the OptFlux platform. The processor of PC is i7-6700 CPU with 3.40GHz frequency, and the RAM is 16.0GB.

The performance of OptRAM has been compared with OptFlux by three strain design cases in yeast, including production of succinate, 2,3-butanediol, and ethanol. To make a more suitable comparison with a previous strain optimization method using an integrated regulatory-metabolic model, we compared OptRAM with OptORF, the first strain design method utilizing regulatory information, for ethanol overproduction. We used the integrated *E*.*coli* model iMC1010 by OptORF to simulate the modification strategy for ethanol production with OptRAM. We found the best solution of OptRAM includes knockdown of *Pta*, *TpiA*, *AldB*, *ZntA*, *YbiV*, *Fre*, *OmpL*, *AceA*, and *GpmB*, as well as overexpression of *Acs*. The ethanol production improved 1.8 fold with 7% reduction in biomass compared to wild-type. In comparison, OptORF suggested deletion of *ArcA*, *Pta*, *TpiA*, *EutD*, *and PtsH*, and overexpressing gene *Edd*, which was predicted to improve ethanol production by 2.2 fold with 54% reduction in biomass. However, *ArcA* has positive regulation on *AckA*, which is one of the essential genes for *E*.*coli* (from DEG). Hence, this solution from OptORF may cause death of *E*.*coli*. While there are benefits to both approaches, in this case it may be that OptRAM identified a more biologically feasible modification strategy with similar improvement on target production compared to OptORF.

## Discussion

### Integrated regulatory-metabolic network can improve *in silico* strain design

With the development of industrial biotechnology, there is an increasing need to design high producing strains in an economic and efficient manner. Computational strain optimization algorithms have been developed for this purpose as an important application of metabolic network reconstructions and constraint-based modeling. These methods can automatically search for sites of genetic modification for increasing any desired product. However, most strain optimization algorithms can only utilize a metabolic network alone and cannot provide strategies also involving transcriptional regulation. Although some methods can utilize gene regulatory information now, they have some limitations since they are based on integrating a boolean regulatory network, which is not suitable for TF overexpression (e.g. OptORF). Reconstructing such models requires extensive manual adjustment and additional information for generating boolean logic rules in the regulatory network [37], which hinders the ability of these algorithms to be broadly applicable across many organisms. In this study, we developed OptRAM to identify the manipulations of both TF and metabolic genes including overexpression, knockdown and knockout. OptRAM uses the framework of simulated annealing and is based on the integration of an inferred regulatory network with a metabolic network from our previous work (IDREAM) [30]. Through the *in silico* strain design case studies for producing succinate, 2,3-butanediol, and ethanol in yeast, we demonstrated that OptRAM can identify solutions containing both TF and metabolic gene manipulations that are predicted to increase production beyond what is seen currently, or found as potential designs using alternative methods.

### OptRAM outperforms other methods for succinate, 2,3-butanediol and ethanol overproduction

OptRAM used simulated annealing with a novel objective function, which can ensure a favorable coupling between desired chemical production and cell growth. We applied OptRAM in succinate, 2,3-butanediol, and ethanol overproduction in yeast. By setting a loose constraint (at least 50%) and a strict one (99%) to biomass respectively, we compared the flux ranges of target reaction in different mutant models by different methods. In both cases, strategies from OptRAM led to higher minimum fluxes for target production under the strict constraints (Tables 5, 7 and 9) than alternative approaches, which indicated that the target chemical is strongly coupled with growth from the OptRAM designs. However, the minimum predicted output fluxes of target in other models are lower and may even be close or equal to zero, which means the coupling relationships are likely weak. We also found most of the genes predicted to be altered could be matched with the reported gene modifications, and some altered TFs having their target genes validated to improve the desired chemical in LASER database. In particular, we conducted fermentation experiment to validate the predicted deletion of *MDH2*, *BDH1*, and *COX4* has significant improvement on ethanol production. Therefore, OptRAM provides *in silico* predictions of improved strains over other methods tested.

### Comprehensive evaluation of mutant solutions is helpful for rational design

Meta-heuristic algorithms commonly provide several optimized solutions with close objective scores, and it is difficult to select a best one for practical operation, such as OptFlux. Thus, we try herein to give a systematic evaluation for these solutions. First of all, essential genes cannot be knocked out without making growth of the cell impossible, so strategies are filtered according to essential genes with experimental validation in DEG and SGD databases. Then, we estimate the implementation cost by setting a score according to connection distance of shortest paths from critical reactions to the main path. Another factor that is weighed is minimizing the adjustment needed to make from the wild type in order to achieve the optimal designed performance. The latter two quantitative indicators are used to assist our selection, along with the flux value of producing the target compound. For succinate overproduction in yeast, there were three solutions excluded since some essential genes for growth were predicted to be knocked out or knocked down. After eliminating these three solutions, we then selected the best remaining solution with maximum target production, which also has lower score of implementation cost and global flux adjustment (S3 Table). For the case of 2,3-butanediol overproduction in yeast, also three solutions with essential gene knockouts were excluded. Of the other solutions that have the same maximum target production (S3 Table), we selected the solution with the lowest summation of path score and flux variation, which provided more suitable design modifications for real experiment design. For the case of improving ethanol production in *E*.*coli*, OptORF predicted deletion of *ArcA* as the modification site, but *ArcA* positively regulated *AckA*, which is one of the essential genes for *E*.*coli*. While OptRAM will avoid knockout or knockdown of such essential genes to keep better growth and/or viability of the organism.

### Future challenge in computational strain design

Despite the above highlights of our new algorithm, OptRAM can be further strengthened in various ways. First, the performance of integrative regulatory-metabolic modeling for phenotype simulation can be improved by introducing more information such as kinetic parameters to set more precise ranges for critical reactions in a kinetic model. Also, by introducing more potential sites, the solution space for exploration increases sharply. It is also favorable to seek new ideas for optimization framework other than focusing on the branches from previously proposed computational strain design methods [60]. On the other hand, the *in-silico* strain design can be enhanced by the inclusion of other forms of regulation, such as allosteric regulation. A constraint-based method (arFBA) for modeling the contribution of allosteric regulation for flux control in the central carbon metabolism of *E*. *coli* has been reported [61]. Most importantly, the method needs to be validated experimentally in many different situations and cases and these data compiled, so that this method and future methods can be iteratively enhanced. In conclusion, in current situation, OptRAM provides a good solution to assist the biologists to identify strain design strategies for particular applications.

## Supporting information

S1 Table Essential genes for yeast growth in SGD and DEG database. (XLSX)

S2 Table The plasmid and primers used in the yeast experiment. (PDF)

S3 Table The detail description of OptRAM solutions for succinate, 2,3-butanediol, and ethanol overproduction in yeast. There are 10 solutions for each case.
(XLSX)

S4 Table The comparison of predicted mutation sites with reported successful modifications by *in vivo* experiment in LASER database for succinate, 2,3-butanediol, and ethanol optimization. (XLSX)

S1 Script The pipeline of OptRAM. (DOCX)

S1 Code The source code of OptRAM with annotation. (RAR)
