# Supplementary material for: OptRAM: In-silico strain design via integrative regulatory-metabolic network modeling
Source: PLoS Comput Biol. 2019 Mar 8;15(3):e1006835. doi: 10.1371/journal.pcbi.1006835 (PMC6426274; doi:10.1371/journal.pcbi.1006835)
Supplement: S2 Table — (PDF) [file pcbi.1006835.s002.pdf]

S2-Table. The plasmid and primers used in the yeast experiment

Plasmid used in this study

Plasmid	Description	Reference
HO	Kan MX4 resource	
Cas9-NAT	Cas9 protein expression plasmid	Zhang *et al.* 2014
pRS42H-gRNA	gRNA expression plasmid	This study
pRS42H-gRNA_MDH2	gRNA expression plasmid targeting *MDH2* gene	This study
pRS42H-gRNA_COX4	gRNA expression plasmid targeting *COX4* gene	This study

Primers used in this study

Primer	Sequence(5'→3')
BDH1-F	ATGAGAGCTTTGGCATATTTCAAGAAGGGTGATATTCACTGACATGGAGGCCCAGAATAC
BDH1-R	TTACTTCATTTCACCGTGATTGTTAGGCGTCAATAGAATCCAGTATAGCGACCAGCATTC
gRNA-MDH2-F:	GATCAGTGGCTCGACACGGAAATG
gRNA-MDH2-R:	AAACCATTTCCGTGTCGAGCCACT
gRNA-COX4-F:	GATCATCTTTCATGGTACCCTTCC
gRNA-COX4-R:	AAACGGAAGGGTACCATGAAAGAT
MDH2-donor-F	TCAACGGTGTTACCGCCGACTTGTCTCATATAGAC**TAA**CCCATTTCCGTGTCGAGCCACT
MDH2-donor-R	GCAAACAGTTCTCAATGCCACCTGCAGGAGAGTGGCTCGACACGGAAATGGG**TTA**GTCTA
COX4 donor-F	GGTATCGATGTTTTCGACACCAAACCATTAGATTCG**TAA**AGGAAGGGTACCATGAAAGAT
COX4 donor-R	ATAATCATCATAAGATTCAATGATGATCGGATCTTTCATGGTACCCTTCCT**TTA**CGAATC

Note: The homologous sequence was characterized as underline, the introduced stop codon (TAA) was presented in bold characters.
