# Supplementary material for: OptRAM: In-silico strain design via integrative regulatory-metabolic network modeling
Source: PLoS Comput Biol. 2019 Mar 8;15(3):e1006835. doi: 10.1371/journal.pcbi.1006835 (PMC6426274; doi:10.1371/journal.pcbi.1006835)
Supplement: S1 Script — (DOCX) [file pcbi.1006835.s005.docx]

Description:

The OptRAM algorithm takes in a regulatory network and a genome-scale metabolic model as input. The algorithm outputs a solution of strain design for a desired product. The attached algorithm is implemented in MATLAB and requires the GUROBI optimization solver and the COBRA toolbox in MATLAB.

1. Initialization:

(1) Using FVA to remove reactions with lb=ub=0 in metabolic model, as well as corresponding metabolites and genes.

(2) Using pFBA to get the reference flux distribution of wild type.

>>[v,regnet,model] = OptRAM_init(rawmodel,rawregnet)

INPUTS:

rawmodel -- original metabolic model

rawregnet -- original regulatory network, including elements of TFnames (list of transcriptional factors), mgene (list of metabolic genes being regulated) and mat (a matrix of coefficients corresponding to TFs and target genes, where 0 means no regulation, a value larger than 0 means an activating regulation and a value smaller than 0 means an inhibiting regulation).

OUTPUTS:

v -- reference flux distribution of wild-type from pFBA

model -- processed metabolic model without redundant reactions and genes

regnet -- regulatory network with target genes match with genes in metabolic model

2. Main process of optimization:

The main process of optimizing strain designs with simulated annealing algorithm.

>>[new_model,objmax,indmax] = OptRAM_main(model,v,regnet,BPCYids,loc)

INPUTS:

model -- processed metabolic model

v -- reference flux distribution of wild-type

regnet -- processed regulatory network

BPCYids -- a vector including reaction IDs for BPCY formulation, where the first ID should be the reaction of biomass, the second ID should be the exchange reaction of target product, the last ID should be the exchange reaction of major carbon source.

loc -- a dictionary for outputting the log file, default is the current dictionary

OUTPUTS:

objmax -- the score of objective function of final solution from current simulated annealing optimization

indmax -- the array including mutated sites and corresponding operation codes of final solution

a log file--this file records the optimization process, every line records a solution with mutated sites (and corresponding operation codes in brackets) and the score of objective function, or the current temperature in SA if changed. The final line records the final solution as well as its score of objective function, flux value of target product and flux value of biomass.

3. Evaluation

It is suggested to run more than five (recommend 10) processes of simulated annealing. Then use the function ‘multi_check’to evaluate all the solutions. Please run this script under the dictionary including all log files being evaluated.

>>[summary,effectess,xgenes,xrxns,geneids,codes,genes,counttable,fluxes,paths,pathoutput]=multi_check(model,v,regnet,BPCYids,nlog,index2del,essentialgene)

INPUTS:

model -- processed metabolic model

v -- reference flux distribution of wild-type

regnet -- processed regulatory network

BPCYids -- a vector including reaction IDs for BPCY formulation

nlog -- number of log files

Optional INPUTS:

index2del -- indexes of small molecule metabolites in metabolic model, these metabolites should be removed in path analysis

essentialgene -- indexes of essential genes for evaluating solutions

OUTPUTS:

summary -- summary of each solution, including score of objective function, min value and max value of target reaction, flux value of growth, number of metabolic genes, number of TFs and flux variation (Cosine) between mutant and wild-type flux distribution.

xgenes, xrxns -- critical genes that has a major effect of score of objective function in one solution, which means if the mutated gene is excluded from the solution, the score of objective function will reduce apparently, here we use a cutoff of 10% reduction; xrxns are critical reactions that has a major effect of score of objective function in one solution, same cutoff as the critical genes.

effectess -- number of critical genes and number of critical reactions in one solution. If essentialgene is in, number of essential genes being down-regulated in one solution will be output.

geneids, codes -- IDs of mutated sites in model (ID of TF will be the index in regnet.TFnames plus the number of metabolic genes); operation codes of corresponding mutated sites, where 0 means knockout, values larger than 0 means over-expression, values smaller than 0 means knockdown.

genes -- Names of mutated sites

counttable -- count of mutated sites from all solutions

fluxes -- flux distributions from all solutions, where the constraints of each model are determined by the mutated sites and codes.

Optional OUTPUTS:

paths -- summary of path analysis for each solution, where the major path from carbon source to the desired product is searched and branches from the major path to critical reactions are searched. Outputs include length of the major path, minimal flux through the major path, mean length of branches, number of branches and path score

pathoutput -- detailed outputs of path analysis for each solution

Example:

In-silico strain design with succinate as the target product in yeast. All data in mat format can be accessed from the example data.

>> load yeast76 %this file has the yeast7.6 metabolic model

>> load regnet %this file has the regulatory network of yeast from IDREAM

>> [v,regnet,model] = OptRAM_init(yeast76, regnet); %Initialization

>> load BPCY_y76_initout_suc %this file has the BPCYids for succinate in processed yeast7.6 metabolic model

>> [new_model,objmax,indmax] = OptRAM_main(model,v,regnet,BPCYids) %Conduct one process of SA, it is suggested to run more than one (e.g 10) processes.

>> load essential %this file has the indexes of essential genes for yeast (from DEG and SGD) in processed yeast7.6 metabolic model

>> load index2del %this file has the indexes of small molecule metabolites in processed yeast7.6 metabolic model

>> [summary,effectess,xgenes,xrxns,geneids,codes,genes,counttable,fluxes,paths,pathoutput]=multi_check(model,v,regnet,BPCYids,10,index2del,essentialgene) %make sure all log files are under the current dictionary, and they are named in 'log1.txt' 'log2.txt' ... 'log10.txt'
